# Molecular modeling and molecular dynamic simulation of the effects of variants in the TGFBR2 kinase domain as a paradigm for interpretation of variants obtained by next generation sequencing

**DOI:** 10.1371/journal.pone.0170822

**Published:** 2017-02-09

**Authors:** Michael T. Zimmermann, Raul Urrutia, Gavin R. Oliver, Patrick R. Blackburn, Margot A. Cousin, Nicole J. Bozeck, Eric W. Klee

**Affiliations:** 1 Department of Health Science Research, Division of Biomedical Statistics and Informatics, Mayo Clinic, Rochester, MN, United States of America; 2 Laboratory of Epigenetics and Chromatin Dynamics, Gastroenterology Research Unit, Mayo Clinic, Rochester, Minnesota, United States of America; 3 Department of Biochemistry and Molecular Biology, Mayo Clinic, Rochester, Minnesota, United States of America; 4 Department of Physiology and Biomedical Engineering, Mayo Clinic, Rochester, Minnesota, United States of America; 5 Center for Individualized Medicine, Mayo Clinic, Rochester, MN, United States of America; 6 Center for Individualized Medicine, Mayo Clinic, Jacksonville, FL, United States of America; Wake Forest University, UNITED STATES

## Abstract

Variants in the TGFBR2 kinase domain cause several human diseases and can increase propensity for cancer. The widespread application of next generation sequencing within the setting of Individualized Medicine (IM) is increasing the rate at which TGFBR2 kinase domain variants are being identified. However, their clinical relevance is often uncertain. Consequently, we sought to evaluate the use of molecular modeling and molecular dynamics (MD) simulations for assessing the potential impact of variants within this domain. We documented the structural differences revealed by these models across 57 variants using independent MD simulations for each. Our simulations revealed various mechanisms by which variants may lead to functional alteration; some are revealed energetically, while others structurally or dynamically. We found that the ATP binding site and activation loop dynamics may be affected by variants at positions throughout the structure. This prediction cannot be made from the linear sequence alone. We present our structure-based analyses alongside those obtained using several commonly used genomics-based predictive algorithms. We believe the further mechanistic information revealed by molecular modeling will be useful in guiding the examination of clinically observed variants throughout the exome, as well as those likely to be discovered in the near future by clinical tests leveraging next-generation sequencing through IM efforts.

## Introduction

The transforming growth factor-β (TGFβ) superfamily of signaling proteins is comprised of a diversity of TGFβ receptors, TGFβ ligands, activins, inhibins, and bone morphogenic proteins which collectively regulate a broad spectrum of biologic functions including wound healing, cellular differentiation, and deposition of extracellular matrix proteins [1–3]. Given their role in mediating embryonic development and maintaining the homeostasis of most tissues, the proper function of these signaling proteins is vital for all multicellular organisms. Genetic variants within these molecules or the downstream proteins that mediate and integrate their signals have been shown implicit with human disease including developmental disorders, vascular diseases, and cancer [2, 4–6]. Technological advances in DNA sequencing have fostered a new era of Individualized Medicine (IM), which among other effects is increasing the rate at which new variants in these pathways are being discovered and associated with disease phenotypes [7]. While the total number of known TGFβ family variants has increased, those characterized by experimental information enabling conclusions as to pathogenicity or the lack thereof are substantially fewer. While well designed functional studies provide a high level of confidence in classifying a variant as pathogenic [8], they are typically costly and time consuming, thus limiting wide-spread use to systematically characterize variants of unknown significance (VUSs). Subsequently, a need exists for higher-throughput computational and experimental methods to evaluate the functional impact of variants at the molecular, biochemical, cellular, and organismal levels.

We are exploring the use of structural bioinformatics, molecular modeling, and molecular dynamics simulations to study the potential mechanisms by which disease-associated missense variants may affect proteins that belong to the TGFβ superfamily. These computational tools leverage three-dimensional protein structures, the protein’s ability to form complexes, and the dynamic behavior of proteins. Methodologically, computational molecular biophysics and biochemistry take advantage of well-validated parameter-based mathematical models, the strengths and weaknesses of which are under continuous evaluation [9, 10] and their potential for translational value has been previously noted [11]. The combination of experimental studies with molecular modeling and molecular dynamics simulations has led to progressively greater understanding of kinase domain functionality at atomic resolution and the role that each residue plays in the native structure [12–15]. We apply lessons learned from these studies about kinase family structure and dynamics to focus our computational analyses. We believe the application of these methods can augment current methods for variant characterization and advance our understanding of the functional impact of sequence variation in members of the TGFβ superfamily.

We leveraged experimental structures of homologous proteins to develop an atomic protein model of TGFBR2 and used it to evaluate the impact of 57 previously identified missense variants. We performed ligand-docking, *in silico* mutagenesis, and molecular dynamics simulations, which extended our understanding of the mechanisms by which different variants affect the TGFβR2 kinase domain. Popular genomics-based predictors (e.g. SIFT [16] and PolyPhen2 [17]) provide predictions of whether or not a DNA mutation is damaging to the function of the encoded protein, while structure-based predictions test the protein structure for specific mechanistic alterations. The time-dependent, three-dimensional dynamic behavior that they reveal adds value to sequence-based computational methods and allows more detailed inference and mechanistic predictions to be made. We propose functional mechanisms for many variants by benchmarking them against the structural and dynamical patterns observed for clinically benign variants. Many of the variants studied are of uncertain clinical significance, some of which alter TGFBR2 similarly to the extent observed for pathogenic variants. Our combination of *in silico* analyses demonstrated utility for understanding previously reported variants that affect the function of this kinase and cause human diseases. We are optimistic that the computational approach presented here improves computational predictions of function and can be useful in characterizing VUSs that will be discovered through clinical testing.

## Results

### Model development

**Fig 1:**
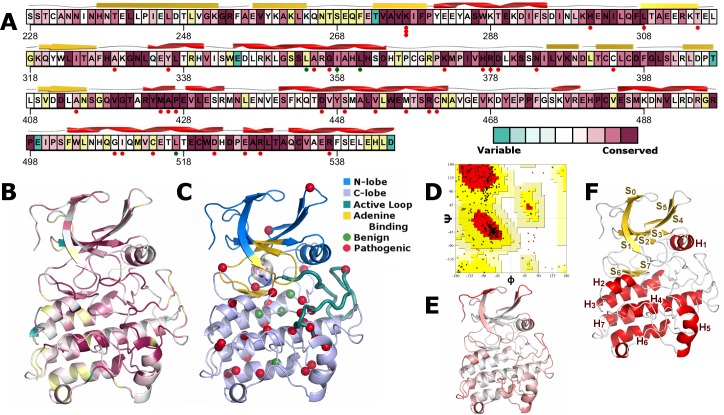
TGFBR2 kinase domain sequence diversity and pathogenic associations summarized along the linear sequence and our structural model. **A)** The background color of the canonical sequence is shown, indicating extent of conservation across paralogs. Amino acid positions with known pathogenic mutations (n = 30) are marked by red circles and those with benign alterations (n = 4) in green. The protein secondary structure from our model is displayed above the sequence. **B)** Coloring the 3D structural model by sequence conservation is more informative than the linear sequence as the regions of conservation have spatial relationships. **C)** The kinase domain consists of two sub-domains; the N- and C-terminal lobes. The adenine binding site lies within a cleft between them. The locations of the 65 variants studied here are marked by spheres at each residue’s C^α^ position. Sites are colored red if the variant(s) at the site is annotated as pathogenic in ClinVar, HGMD, or UniProt. If it is annotated as benign by the same sources, or is manually chosen as a control, we color the site green. Sites with multiple annotations, or only disease phenotype associations, are colored orange. **D)** We validate the quality of our structural model using multiple algorithms (see Methods) including Ramachandran analysis; > 95% of residues within allowed regions. **E)** Overall model quality is evaluated on a per residue basis (e.g. Ramachandran outliers) by QMEAN with residues with a score of ≤ 1 colored in white and scaled linearly to red at a score of 5.8. **F)** Our TGFBR2 model adopts the typical kinase domain architecture. The N-lobe is primarily comprised of a sheet of 5 strands, while the C-lobe is mostly helical.

Significant homology exists between type II and type I TGFβ superfamily receptor kinases [18]. Furthermore, many sequence and structural features of these kinases are deeply conserved, as distantly as bacteria. Thus, the evolutionary relationships among these proteins can be drawn upon for inference on the function of a distinct family member. Our TGFBR2 model was informed by the annotated multiple sequence alignment between the TGFBR2 kinase domain and human paralogs (S1 Fig). Ramachandran plots revealed that 97% of residues were in favored and allowed regions. We considered residues to be of poorer quality if they were outside of the allowed regions in Ramachandran space or in the 95^th^ percentile of QMEAN. These residues are primarily within the N-terminal 15 amino acids, the 4 amino acid surface-exposed loop between strands S4 and S5, and the 7 amino acid surface-exposed loop proceeding helix H5 (Fig 1E). In full-length TGFBR2, the N-terminal residues in our model would connect to the transmembrane helix [19, 20] and their poorer scores may indicate that they adopt a different configuration near the membrane. Surface exposed loops tend to be flexible and change their atomic configuration with relative ease in solution. Thus, the single configuration scored for model quality is less representative of the solution state for these residues. Thus, multiple structure evaluation metrics explain characteristics of our model and indicate that it is of high quality.

### Protein architecture

The kinase domain architecture is organized into two subdomains commonly referred to as the N- and C-lobes (Fig 1). The N-lobe is primarily comprised of beta-strands and the C-lobe of alpha-helices. The first helix within the structure is the only helix in the N-lobe and is referred to as the αC-helix. The position of this helix is an important regulatory component of the kinase. At the interface between the N- and C-lobes is a pocket where ligands bind. ATP is the major physiologic ligand of TGFBR2 and supplies the phosphate for transfer to the target. This process is facilitated by the active site or activation loop, found at the interface of the N and C-lobes. These features play the predominant roles in controlling substrate access.

The entire TGFBR2 protein exhibited high sequence conservation and certain regions were invariant across paralogs. Along the linear sequence, these appeared to be disjointed. After they were mapped to the structural model and their dynamic effects calculated, their functional role was more readily interpretable. Invariant residues were within three regions. The first region consists of residues interacting between helices 5–7, likely preserving the integrity of the C-lobe. The αC helix, within the N-lobe, at the interface between the two domains, was the second region. The third was comprised of the central β-strands within the N-lobe and formed the “ceiling” of the ATP binding site.

**Fig 2:**
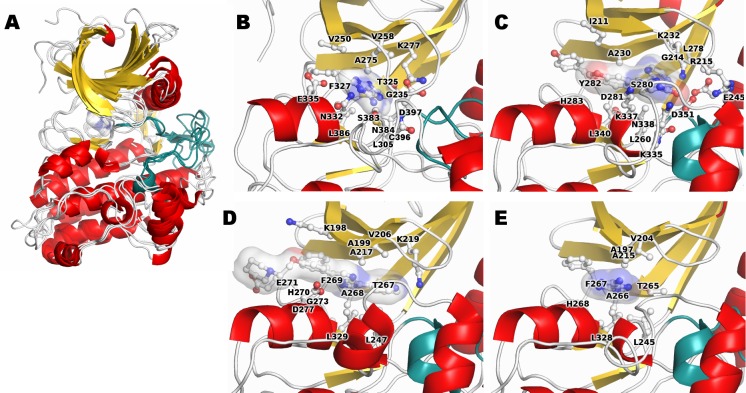
Ligand binding site characteristic for TGFBR2 and paralogs. **A)** Our TGFBR2 kinase domain model is superimposed on the experimental structures of 3 paralogs (TGFBR1, ACVR2A, and ACVR2B), emphasizing the consistency of this structural domain across the family. Each is colored by secondary structure elements, and the active site loop (from the DFG to the MAP sequence motifs; see Methods) in teal. The molecular surface of adenine from our TGFBR2 model is shown. **B)** Adenine binding site from our TGFBR2 model. Residues from both the N- and C-lobes make up the active site. Side chains closely interacting with the bound adenine are shown in detail. **C)** X-ray structure of TGFBR1 bound to an antitumor agent (3tzm). **D)** X-ray structure of ACVR2A with a different antitumor agent bound (3q4t). **E)** X-ray structure of ACVR2B with adenine bound. There are strong similarities to the core of the binding sites across paralogs.

We compared details of the ATP binding site in our model to three human paralogs (Fig 2) in order to assess our model. To evaluate the quality of the docked pose, we compared the residues surrounding our final docked ligand pose with residues in drug inhibitor-bound crystal structures of paralogs. The nucleoside was oriented with its phosphate acceptor groups pointing to the activation loop, a structural and functional feature conserved among members of the kinase superfamily. Further, physiologically critical amino acids were positioned appropriately. For example, the catalytic lysine, K277, is analogous to K232 in TGFBR1 and K219 in ACVR2A; all were found in similar positions relative to their respective ligands. The adenine-binding site is primarily composed of hydrophobic amino acids from the N-lobe, and a mixed composition of hydrophobic and charged amino acids from the C-lobe. These differences in surface properties are similar across the paralogs and likely help to position the ligand properly within the pocket.

### Domain motions from a coarse-grained model

**Fig 3:**
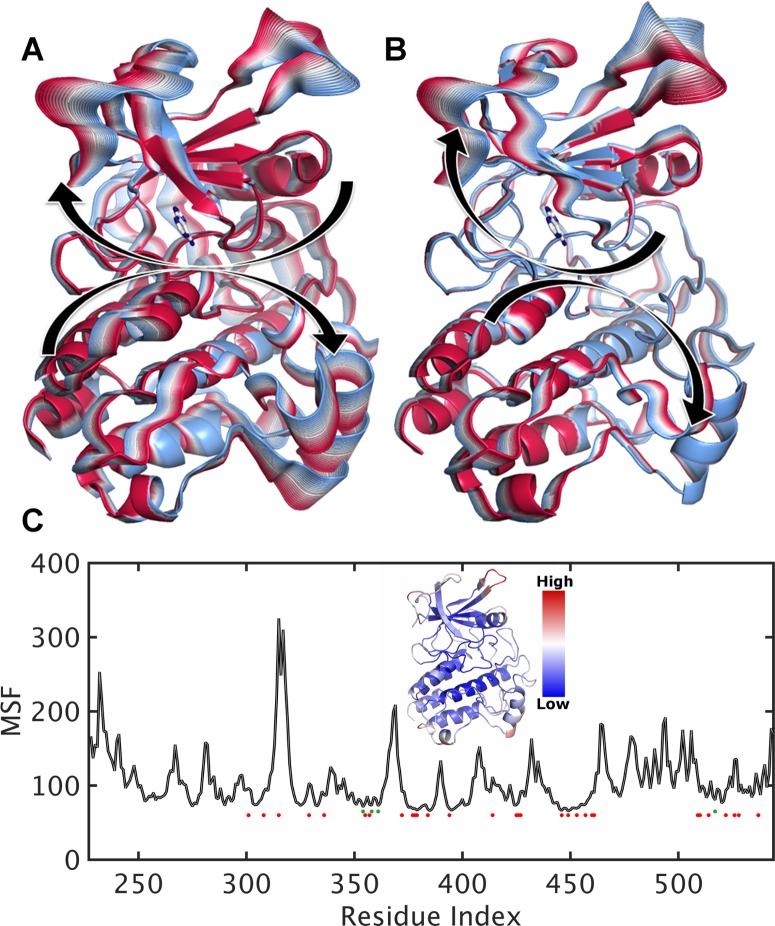
Canonical motions of the kinase domain architecture reveal sites important for functional motions. A) The first mode of motion, or the least energetically taxing way that the kinase domain moves, corresponds to a twisting of the lobes relative to one another. B) The second mode corresponds to a coupled twisting and hinging of the lobes. C) The mobility of each amino acid within the structure can be summarized by Mean Square Fluctuation (MSF), computed from the same model. We plot the MSF of each residue, indicating sites of pathogenic mutations (red points) and benign (green). The inset shows the MSF on the 3D structure.

We began our dynamic evaluation of TGFBR2 using an Anisotropic Network Model (ANM). This model demonstrated twisting and rocking of the N- and C-lobes, with respect to one another (Fig 3). These motions affected the space within the adenine-binding site and above the activation loop and likely reflect functional motions important for the phosphorylation cycle. The regions of the structure with highest flexibility were the same as those identified as potentially lower quality (compare highest QMEAN residues in Fig 1E to those with greatest motion in Fig 3). [19, 20]As many structural evaluation metrics are developed using patterns observed for static representations of high-resolution structures, there is the potential that they are less reliable for highly flexible regions. Therefore, an understanding of the large-scale domain motions provided context for model quality scores and also greater resolution concerning the potential role of each residue in the phosphorylation cycle.

### Atomic molecular dynamics

MD simulations provide time dependent behavior of the molecule in greater detail than ANM modes. We performed MD simulations of 57 variants, comprised of pathogenic variants (n = 30), benign alterations (n = 4), and VUSs (n = 23). Simulations were monitored by RMSD to the initial WT conformation in order to evaluate overall stability. The time-dependent trajectories of each amino acid in each simulation were studied geometrically and energetically.

We first evaluated alterations to K277 which is a critical residue in the phosphorylation cycle. Four different variants have been previously reported and are studied here: K277R/E/D/A. For example, K277R has been used as a model for inactive TGFBR2 [21]. The molecular dynamic simulations of each K277 variant showed effects in the architecture of the adenine binding residues such that fewer hydrogen bonds are formed throughout the simulation (S2 Fig). K277 forms stable hydrogen bonds with D397 and E290 (S3 Fig). These interactions are lost upon mutation, leading to altered dynamics throughout the N-lobe, adenine binding pocket, and active site. For example, the inter-strand hydrogen bonding interactions between D247 and K260 were less occupied in K277 variants, while inter-strand hydrogen bonds between A261 and V274 were stabilized. From this case study of a well-annotated functional variant, we validated our model and procedure as a useful tool for evaluating the full set of disease-associated variants.

### Geometric and energetic evaluation

Pathogenic variants are partially clustered throughout the sequence and tertiary structure (Fig 1) at conserved amino acid positions. Further, apart from K277, no obvious hotspots of pathogenic variants are evident. Thus, identification of how each variant alters the structure and the mechanism by which it may (or may not) be pathogenic is of interest. We focus next on how variants may affect a series of structure and dynamics-based features including: 1) energetic stability, 2) ligand binding site dynamics, 3) activation loop dynamics, 4) flexibility around the variant site, 5) distance between the αC-helix and the activation loop, and 6) alterations in hydrogen bonding. From the benign simulations (WT and 4 benign variants as negative comparators), we identified WT-like thresholds for each metric and labeled a variant as “altered” with respect to each metric when they exceeded the value observed in these benign simulations.

#### Energetic stability

**Fig 4:**
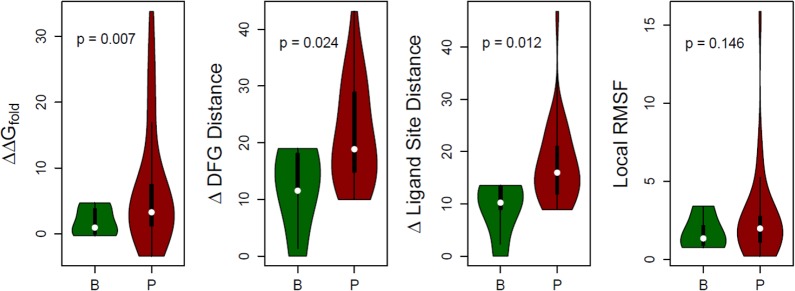
Structure-based evaluations were used to evaluate benign (B) and pathogenic (P) mutations. In these comparisons, benign simulations (n = 5; 4 benign variants and WT) act as negative controls. Variants within each group are summarized by a combined boxplot and density plot where width smoothly scales by the number of variants at each level of the score. **A)** The increase in folding energy upon mutation, ΔΔG_fold_, is greater for many pathogenic variants, compared to benign. **B)** Changes in the DFG structural motif tend to be larger in pathogenic variants, compared to benign and **C)** using the ligand binding site. **D)** A small number of variants lead to increased local fluctuations.

Each variant was generated *in silico*, refined to fix any unfavorable interactions, and stability evaluated and reported as ΔΔG_fold_. Refinement provides more accurate and reliable estimates since the protein molecule may naturally adjust internally to the presence of the variant. Because the TGFBR2 kinase domain is highly conserved, there are few polymorphic variants to act as negative controls. We utilize the WT simulation and 4 benign variants as benign/negative comparators. Comparison between the stabilities of benign and pathogenic simulations reveals that a group of pathogenic variants are highly destabilizing (p = 0.007; see Fig 4).

#### Ligand binding site

**Fig 5:**
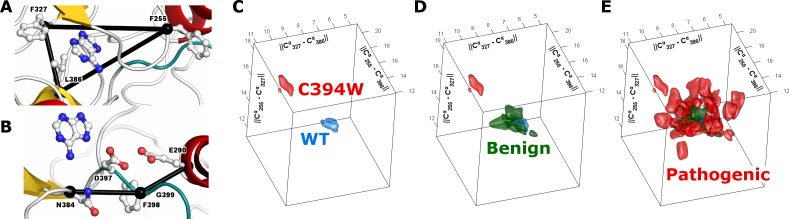
Ligand binding site and active site loop conformational dynamics. We choose representative sites on each side of the ligand-binding site. The distances between these sites are used as monitors of the conformation of each site. We analyzed the direct and allosteric effects of variants on these and other sites. **A)** The C^α^ atoms of residues around the ligand-binding site include F327 “above” the ligand, L386 below, and F255 “across from” the ligand, within the p-loop. **B)** We used C^α^ distances as summary metrics for the DFG conformation: N384, F398 in the center of the motif, and E290. **C)** For the active site distances, the three monitors give a point in a 3D space for each conformation. As the MD simulations progress, we generate a collection of such points, from which a 3D volume is generated that encompassed the densest region of data points, for each variant. The surfaces enclosing half of the sampled distances for our WT simulation, and an example pathogenic variant, C394W, are shown. The separation between the two indicates their conformational differences during our simulations. **D)** Benign variants have little effect on ligand binding site dynamics; the volumes spatially overlap each other and the WT simulation. **E)** Superposition of all pathogenic variants studied shows a wide range of conformational effects.

**Fig 6:**
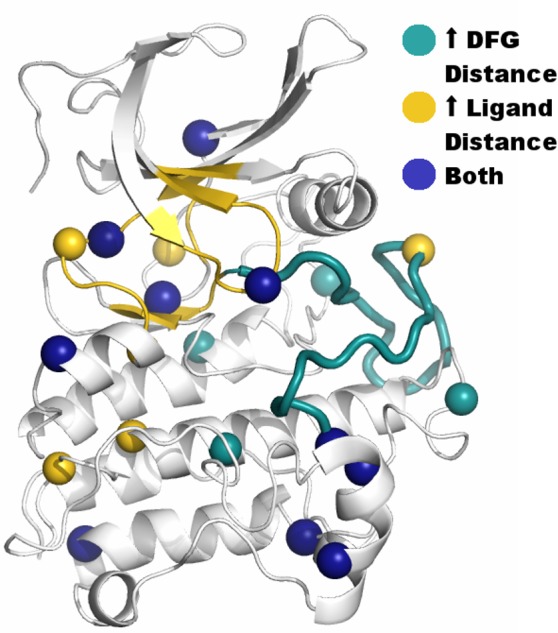
Variants that are distant from the activation loop or the ligand binding site affect dynamics at these sites. Variants that resulted in increased dynamics either the activation loop or the ligand binding site are indicated by spheres at their C^α^ atom position. The activation loop and ligand binding site are highlighted as in Fig 1. We defined an increase by values greater than those observed in benign simulations. Residues that when mutated alter dynamics at these sites are distributed throughout the structure.

Dynamic changes in the ligand binding site, where ATP binds, were monitored for each variant using 3 reference amino acids (Fig 5). The C^α^ atom positions of these residues around the ligand-binding pocket are used to monitor its overall conformation: F327 “above” the ligand, L386 “below”, and F255 “across from” the ligand within the p-loop. These three distances are used to define the normal geometry of the active binding site and can be represented three-dimensionally. Each simulation is visualized as a volume in this three-dimensional space (Fig 5) and differences between variants quantified by their separation. A subset of pathogenic variants appear to affect ATP binding and thus impair the function of the TGFBR2 kinase domain (p = 0.012; Fig 4). Amino acid variants throughout the structure were shown to affect dynamics in and around the ligand-binding pocket (Fig 6). We also measured the distance from these three reference points and a bound adenine, and show that the differences in the pocket geometry lead to differences in ligand positioning (S4 Fig). Therefore, pathogenic variants may affect the ATP binding site conformation and/or dynamics directly or indirectly.

#### Activation loop

The dynamic flexibility of the activation loop across related kinases is regulated by phosphorylation, is important for the appropriate positioning of catalytic residues, and controls the substrate’s access to the catalytic site. The mechanical positioning of these components has been shown necessary to either endow or deprive TGFBR2 of its kinase activity. Substitution of amino acids in and around the activation loop may affect these dynamics. We assessed changes in the activation loop conformation by recording two distance monitors (Fig 5) using a similar approach described for the ligand-binding pocket monitoring. Pathogenic variants were more likely to alter the conformation of this structural region than benign variants (p = 0.024; Fig 4). Further, amino acid variants throughout the structure, not just those within the vicinity, were shown to affect dynamics at the activation loop (Fig 6). These analyses provided mechanistic information on the potential contribution of each variant to the dynamics of the activation loop and regulation of the ATP binding site.

#### Flexibility around the variant site

We measured structural flexibility around the altered site, defined as the RMSF (see Methods) of an 11 amino acid window centered on the site. This analysis is a local measure of the dynamic changes induced by the variant. While the difference between groups was not statistically significant (p = 0.146), some pathogenic variants induced markedly increased local dynamics (Fig 4). Therefore, some variants’ functional consequence may be to locally destabilize the structure, potentially leading to altered function or interactions with other proteins.

#### Position of αC-helix and activation loop

Amino acids pack together in specific ways to assemble signaling networks within the structure and these networks have been shown critical to enzyme function and specifically to the transition between active and inactive states [15, 22, 23]. The relative position between the αC-helix and the activation loop is an indicator of this transition. Pathogenic variants were more likely than benign to favor increased separation (p = 0.093) and thereby greater substrate accessibility. Thus, some pathogenic variants may result in a bias for the active conformation by influencing the relative positioning of these structural elements.

#### Alterations in hydrogen bonding

For each variant, we identified the hydrogen bonds present and summarized them at the residue level–which pairs of residues interact via hydrogen bond(s) and for what fraction of time (S2 Fig). Many variants introduce new interactions via alterations in the hydrogen bond network or abolish interactions that are typically present. Specific hydrogen-bonded interactions within the kinase architecture have been previously studied and their alteration identified as functional [24, 25]. Therefore, changing of the hydrogen-bond network is another means by which variants may alter (restrict activation/inactivation switch) kinase function.

#### Application of 3D information to VUS interpretation

**Fig 7:**
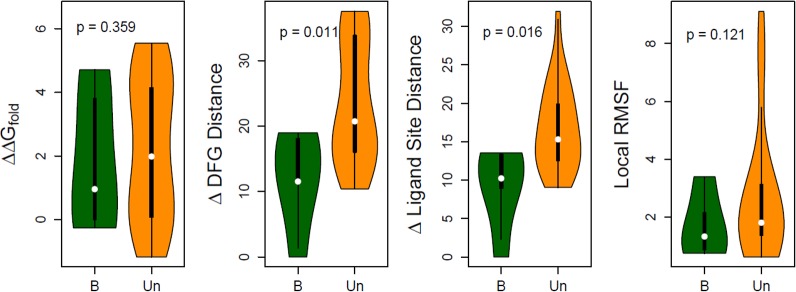
Application of structural metrics to simulations of observed variants with unknown functional consequences. Many variants of uncertain significance, with conflicting annotations, or individual reports of disease associations, show alterations in structural features.

**Fig 8:**
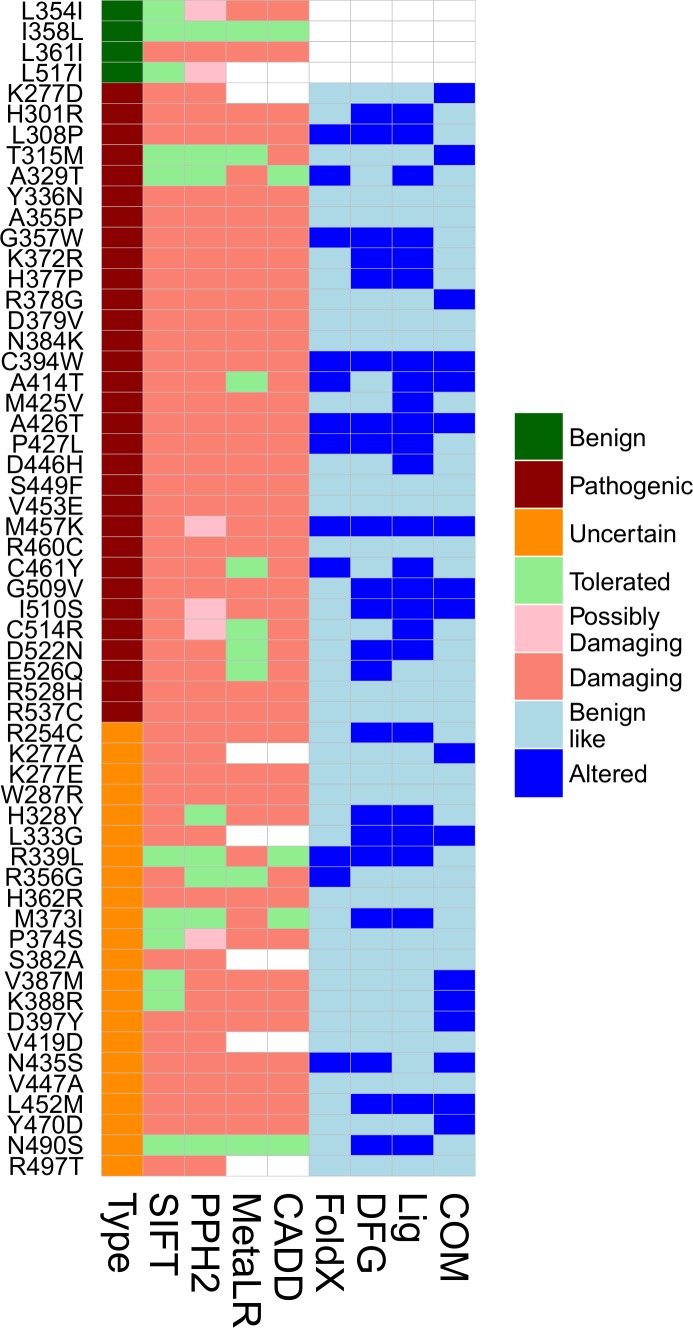
Description of TGFBR2 variants using genomics-based and structure-based evaluations. The same data as is presented in Table 1 is shown graphically. Genomics-based predictors provide predictions of damaging, while structure-based predictions test for specific mechanistic alterations.

Discrete scores for each structure-based metric were used to determine which variants altered one or more feature leading to a mechanistic interpretation of the variant’s effect, and how this information augmented available genomic-based predictive algorithms. First, the structure-based metrics were applied to a set of VUSs (n = 23), which revealed that many VUSs lead to dynamic changes (Fig 7) similar to pathogenic variants. Genomics-based predictive algorithms classified the majority of VUSs as damaging to the protein, but don’t provide information about functional consequence or mechanism by which they are damaging (Table 1 and Fig 8). From our simulations, we assigned a functional alteration(s) to 71% (22/31) of pathogenic variants and 64% (14/22) of VUSs. Thus, gains were achieved for both types–greater information was provided for many of the pathogenic variants, while greater evidence is gathered to potentially promote or demote the VUSs.

**Table 1:** Description of TGFBR2 variants using genomics-based and structure-based evaluations.

			Genomics-Based				Structure-Based			
Var	Type	ExAC^‡^	SIFT^†^	PPH2	MetaLR	CADD	ΔΔG_fold_	ΔDFG	ΔLig	ΔCOM
L354I	Benign	8.2x10^-6^	B	poD	D	20.8				
I358L	Benign	0	B	B	B	10.5				
L361I	Benign	0	D	prD	D	28.6				
L517I	Benign	0	B	poD	NA	NA				
K277D	Pathogenic	0	D	prD	NA	NA	0	0	0	1
H301R	Pathogenic	0	D	prD	D	25.5	0	1	1	0
L308P	Pathogenic	0	D	prD	D	27.3	1	1	1	0
T315M	Pathogenic	3.0x10^-3^	B	B	B	22.8	0	0	0	1
A329T	Pathogenic	5.8x10^-5^	B	B	D	15.8	1	0	1	0
Y336N	Pathogenic	0	D	prD	D	27.5	0	0	0	0
A355P	Pathogenic	0	D	prD	D	27.4	0	0	0	0
G357W	Pathogenic	0	D	prD	D	32.0	1	1	1	0
K372R	Pathogenic	0	D	prD	D	26.7	0	1	1	0
H377P	Pathogenic	0	D	prD	D	25.3	0	1	1	0
R378G	Pathogenic	0	D	prD	D	25.8	0	0	0	1
D379V	Pathogenic	0	D	prD	D	27.9	0	0	0	0
N384K	Pathogenic	0	D	prD	D	24.2	0	0	0	0
C394W	Pathogenic	0	D	prD	D	26.3	1	1	1	1
A414T	Pathogenic	0	D	prD	B	32.0	1	0	1	1
M425V	Pathogenic	0	D	prD	D	27.3	0	0	1	0
A426T	Pathogenic	0	D	prD	D	34.0	1	1	1	1
P427L	Pathogenic	0	D	prD	D	34.0	1	1	1	0
D446H	Pathogenic	0	D	prD	D	34.0	0	0	1	0
S449F	Pathogenic	0	D	prD	D	34.0	0	0	0	0
V453E	Pathogenic	0	D	prD	D	34.0	0	0	0	0
M457K	Pathogenic	0	D	poD	D	33.0	1	1	1	1
R460C	Pathogenic	0	D	prD	D	35.0	0	0	0	0
C461Y	Pathogenic	0	D	prD	B	28.8	1	0	1	0
G509V	Pathogenic	0	D	prD	D	31.0	0	1	1	1
I510S	Pathogenic	0	D	poD	D	32.0	0	1	1	1
C514R	Pathogenic	0	D	poD	B	25.8	0	0	1	0
D522N	Pathogenic	0	D	prD	B	31.0	0	1	1	0
E526Q	Pathogenic	0	D	prD	B	29.0	0	1	0	0
R528H	Pathogenic	0	D	prD	D	34.0	0	0	0	0
R537C	Pathogenic	0	D	prD	D	35.0	0	0	0	0
R254C	Uncertain	0	D	prD	D	34.0	0	1	1	0
K277A	Uncertain	0	D	prD	NA	NA	0	0	0	1
K277E	Uncertain	0	D	prD	D	28.3	0	0	0	0
W287R	Uncertain	0	D	prD	D	27.5	0	0	0	0
H328Y	Uncertain	0	D	B	D	21.0	0	1	1	0
L333G	Uncertain	0	D	prD	NA	NA	0	1	1	1
R339L	Uncertain	0	B	B	D	19.6	1	1	1	0
R356G	Uncertain	0	D	B	B	24.4	1	0	0	0
H362R	Uncertain	0	D	prD	D	24.5	0	0	0	0
M373I	Uncertain	0	B	B	D	13.6	0	1	1	0
P374S	Uncertain	0	B	poD	D	24.0	0	0	0	0
S382A	Uncertain	0	D	prD	NA	NA	0	0	0	0
V387M	Uncertain	1.1x10^-3^	B	prD	D	24.5	0	0	0	1
K388R	Uncertain	0	B	prD	D	22.5	0	0	0	1
D397Y	Uncertain	0	D	prD	D	29.2	0	0	0	1
V419D	Uncertain	0	D	prD	NA	NA	0	0	0	0
N435S	Uncertain	0	D	prD	D	27.9	1	1	0	1
V447A	Uncertain	0	D	prD	D	28.6	0	0	0	0
L452M	Uncertain	0	D	prD	D	28.5	0	1	1	1
Y470D	Uncertain	0	D	prD	D	33.0	0	0	0	1
N490S	Uncertain	0	B	B	B	14.4	0	1	1	0
R497T	Uncertain	0	D	prD	NA	NA	0	0	0	0

^†^ D, Damaging; B, Benign; prD, probably damaging; poD, possibly damaging; NA, not applicable. We mapped each protein variant to all DNA variants that could generate it, and report here the most impactful of the DNA changes.

^‡^ Allele frequency in the ExAC database.

## Discussion

We aim to gain insights into the effects of amino acid variants on the TGFBR2 kinase domain and to provide mechanistic interpretations. Using a molecular model of the protein structure to predict changes in stability and dynamic behavior upon mutation, we present the case for greater application of these methods. Hypothesizing that variants leading to more severe structural effects will be evidenced by alterations in folding energy, local flexibility, regulatory loop positioning, or loss of important structural contacts including ligand binding site conformation, relatively short simulations were used. We believe that the widespread adoption of these methods to the prioritization and interpretation of clinically observed variants within the context of IM initiatives is likely to have a significant positive impact on the biomedical community.

We have applied a series of 3D structural and dynamical evaluations to simulations of variants within the TGFBR2 kinase domain in order to gain a greater resolution on the molecular effects of VUSs than is currently available from standard genomics-based predictive algorithms. We have shown that understanding domain motions provides context for each residue’s role in the phosphorylation cycle. Comparison of global stability metrics revealed that a group of pathogenic variants were highly destabilizing. Pathogenic variants directly or indirectly affected the ATP binding site, were more likely to alter the conformation of the activation loop and its position relative to the αC-helix, or altered the internal hydrogen-bond network. Any of these alterations could potentially lead to alteration or deregulation of TGFBR2 function. Using these observations of the impact of pathogenic variants on the TGFBR2 protein as a benchmark, the resolution with which VUSs in the kinase domain of TGFBR2 can be functionally interpreted was improved.

Increased functional resolution of VUS effects will be clinically valuable when alterations of one type have different therapeutic implications than another, such as distinguishing between variants that lead to loss of stability from those leading to constitutive activation. Our work reports the development and validation of a model for the TGFBR2 kinase domain that can be used in conjunction with experimental structures (e.g. those of human paralogs) to gain insight into the potential effect of disease-relevant variation. This model can be used to infer the potential effects of previously described and newly observed variants in the TGFBR2 kinase domain on the enzyme’s function, which may affect the prioritization of functional assays or treatment decision-making. For example, activating variants could be inhibited, while destabilizing variants could require a different therapeutic approach. As increasing numbers of novel variants are emerging from IM initiatives and NGS-based clinical tests, efforts such as the American College of Medical Genetics guidelines for interpretation of variants are providing standard methods for results interpretation [26]. However, new methods for evaluating the impact of sequence variation on protein structure and function are needed in order to achieve greater resolution. Advancements and methods such as the ones described in this paper may provide an additional line of evidence to be considered during variant interpretation and have the potential for significant translational value. These methods represent an analysis paradigm that has been used in basic research, and has emerging value for translational and clinical sciences.

Molecular modeling is dependent on availability of or ability to generate robust protein models. TGFBR2 has no experimental structure, but homology to extant structures was sufficient to generate multiple high-quality models. This level of detail has already been shown to add value over sequence-based methods [27, 28], for example the 3D convergence of sequence-disjoint observations also known as 3D hotspots [29]. Algorithms used in high-throughput settings for interpreting or prioritizing variants are limited to static structural models, but we have demonstrated that additional information guiding the interpretation of a variant can be derived by also considering dynamic effects. Here we refine and animate each model using physics-based simulations and used these to evaluate structural and dynamic features for a set of benign, pathogenic, and VUSs in TGFBR2.

It is well established that protein sequences typically contain all necessary information needed to encode a 3D structure, that the 3D structure encodes functional dynamics, and that the combination of the structure and its functional dynamics are often necessary for biologic processes [30–35]. Proteins are not static entities, but are flexible biomolecules that continuously undergo rearrangements in response to their environment or interactions with other molecules. Many computational biophysical methods have been developed to model the dynamics of protein structures including Normal Mode Analysis (NMA) and Molecular Dynamic (MD) simulations. Here we employed a type of NMA, the Anisotropic Network Model (ANM) [36] to determine a set of canonical motions for TGFBR2. These motions are ordered by how easy it is for the structure to “deform” by them. MD is a time-dependent simulation of motion that takes into account the physicochemical details of protein’s atomic structure. The primary output of MD is the detailed positional and energetic data from the time-dependent simulation. Interestingly, the dominant motions computed from MD are often similar to modes calculated by NMA. Thus, the two methods can provide different points of view on molecular motion: NMA is a computationally efficient method for determining large-scale or collective motions, while MD provides detailed, time-dependent dynamics, and identification of energetic contributions to molecular motion. Importantly, any mutations that affect the ability of the structure to achieve these motions would impact functional dynamics.

Recent reviews have emphasized characteristics of the kinase family including the critical mechanistic roles of many of the amino acids [15] in determining and transmitting functional dynamics. The regulatory and catalytic spines are structural features of conserved hydrophobic amino acids that act as communication channels between the N and C-terminal lobes. They connect the αF helix (H4) to the αC helix (H1) and coordinate the conformational changes necessary for the active to inactive conformational switch. This is further coordinated with the activation loop, or A-loop, which is phosphorylated in many kinase families to further drive the switching behavior. These conformational changes regulate accessibility of the adenine-binding site, positioned between the two lobes. These large-scale motions of the protein are recapitulated in our ANM model and within MD simulations. They are the basis behind the “action at a distance” that we observe by variants throughout the structure, which lead to dynamic effects at the ligand binding site and activation loop.

In many clinical settings, causation is implied by repeated observation. That is, when multiple patients with the same phenotype have samples sequenced and a common position of mutation (hotspot) is observed, it is often concluded that it is either the causal mutation or a driver mutation [37, 38]. However, in many cases, private or novel variants are discovered, or the observed variant was seen in association with a phenotype different from the case-at-hand, making inference less direct. Distinguishing nuanced differences between and among variants is the primary advantage of structure-based metrics as they provide more mechanistic insight into the effect of each. One variant may destabilize the native fold, another may alter dynamics, and a third may prevent association with other proteins or molecules.

It is also important to discuss the predictive value of the current model, and molecular modeling in general, for the interpretation of variants that may be discovered by NGS-based clinical tests, particularly as part of IM efforts. From analyses of our model, we conclude that alterations throughout the structure are capable of affecting the activation loop or ATP binding pocket. This phenomenon is well established in biophysics and is typically referred to as allostery [39–41] or allosteric regulation [42]. The expansion of clinical annotations from the current paradigm of “nearby in sequence” to those alterations that may be nearby in structure or nearby in allosteric distance, will require greater computational complexity, but is likely to enable greater understanding of the effects of variants on protein function. These methods are well established and reliable in cases of at least moderate sequence conservation [9, 43]. While not all proteins will have a structural template, a large fraction of the disease- and therapy-relevant proteins do [44–47] and any current translation of methods from structural biology and computational biophysics to the interpretation of coding variants will be beneficial.

The duration of time that simulations are computed for varies and has a large impact on the probability of observing structural or dynamic differences between conditions. In this work, we have used relatively short implicit solvent simulations that probe how the native structure responds to each variant. Increasing the duration of simulation may also increase the sensitivity with which differences between variants may be identified. Further exploration as to the relative differences between benign and pathogenic alterations based on the choice of simulation duration, extent of minimization, force field, solvation, crowding effects, etc. is warranted and will likely differ based on the protein architecture (globular, fibrous, etc.) and cellular environment (cytosolic, membrane bound, or within organelles).

During preparation of this manuscript, an experimental structure of TGFBR2 kinase domain was released [48]. By comparing this structure to our model, we have confirmed the reliability of our model (S5 Fig). The ligand interacts with the same residues. Four loops are in different positions. There were six charged residues within or nearby these loops that were mutated to alanine in this experimental structure and could have influenced their positioning. The main structure, ignoring these loops, is highly superimposable: 1.295 Å C^α^ RMSD. Further, these loops are the most mobile within our simulations. Thus, the high agreement between our model and this experimental structure, not released until after we had completed our modeling work, confirms our model’s reliability and provides another positive example of the utility of comparative modeling.

The medical value of this work lies in highlighting computational approaches with the ability to provide insight into both the mechanism of disease-associated mutations and evaluation of their potential pathogenicity. Current clinical paradigms focus on the identification of missense alterations using DNA-based tests and without thorough consideration of the three-dimensional and dynamic biomolecule. Protein structure modeling provides for a more detailed understanding of the potential effects of missense variants. In the current study, we validated our model with several well-characterized pathogenic variants, and evaluated a collection of VUSs. Our approach can inform the interpretation of variants, by providing possible mechanisms of functional alteration and by demonstrating greater evidence to promote or demote VUSs. We anticipate that our TGFBR2 model and the generalization of this approach to other proteins of interest will be useful for the future characterization and functional interpretation of novel disease-associated variants.

## Conclusions

The interpretation of novel variants in the TGFBR2 kinase domain is important for furthering our understanding of several human diseases. This task has increased in scope due to the widespread application of clinical next generation sequencing, which is uncovering disease-associated variants in many proteins at a faster rate than ever before. Consequently, in this work, we evaluated the utility of short MD simulations for assessing the potential impact of variants, revealing various mechanisms by which they may lead to functional alteration. Our results also underscore that the function most likely affected by each variant may be allosteric in nature. Differentiating which variants may lead to dysfunction and the mechanism underlying these alterations is not possible from current sequence-based analysis. Therefore, we believe that the mechanistic information revealed by molecular modeling will be critical for the examination of variants discovered by clinical sequencing tests, particularly for individual patient cases as resulting from ongoing IM efforts. Hence, we are optimistic that the methodology and information gathered in this study will have clinical utility.

## Methods

### Molecular modeling

We began from the TGFBR2 canonical UniProt sequence for P37173-1, and mapped to Ensembl transcript ENST00000295754 for linking to genomic annotations and paralogs. Because no experimental structure of TGFBR2 exists, known structures of homologous sequences were chosen based on sequence homology computed by T-Coffee alignment [49] and BLAST queries to the PDB [50] using the non-redundant human reference [51, 52]. An appropriate structural template with 46% sequence identity for the modeled region was identified in ACVR2B. The 3D structure of the TGFBR2 kinase domain was determined by homology modeling using MODELLER [53] version 9.15 and the ACVR2B-Adenine complex, 2QLU [54] as a template. Ligand docking followed to form the complex (see below). The following modifications were made to the template: (i) addition of hydrogen atoms; (ii) protonation or deprotonation of the Arg, Lys, Asp, Glu and His residues; (iii) energy minimizations of the added hydrogen atoms. The protonation states of all ionizable residues (Arg, Lys, Asp, Glu and His) were determined at pH 7.4 using Discovery Studio [55]. Arg and Lys residues were protonated, unless located in a hydrophobic environment. We generated 20 refined models, which were ranked according to DOPE energy values [56]. The model with the lowest DOPE score was chosen for further analyses. To estimate the quality of the model, we generated Ramachandran plots (Psi vs. Phi angles plot) using PROCHECK [57]. QMEAN [58] was used to summarize multiple quality metrics at the residue level in order to evaluate if differences in quality clustered on the 3D model. Comparisons of the generated homology models by calculations of their electrostatic potentials, volumes, and accessible surface areas were performed using VADAR version 1.8 [59] and Dali [60] version 3. The resulting TGFBR2-adenine complex was refined by a 2.0 ns molecular dynamics (MD) simulation (see below). Normal Model Analysis was generated using the ANM model [61] with interaction strengths decreasing with the square of C^α^ separation [62].

In order to better understand conservation across the TGFBR2 protein sequence, human paralogs of TGFBR1 and TGFBR2 were identified from the Ensembl database [63] and multiple sequence alignment generated using Clustal [64, 65]. This alignment was annotated according to sequence conservation, physicochemical properties, and secondary structure content, using ConSurf [66] and Clustal. Conservation was mapped to the 3D structure using ConSurf.

### TGFBR2 variants and annotation

57 missense variants were extracted from ClinVar [67], HGMD [68], UniProt [69], and ExAC [70], and mapped to our TGFBR2 model along with additional control variants. Variants were classified as pathogenic by ClinVar and HGMD. “Likely” or “suspected” pathogenic variants were classified as VUSs. Variants with conflicting reports in ClinVar were also considered VUSs. All variants in the TGFBR2 kinase domain that were classified as “benign” in ClinVar had conflicting reports; indicated likely pathogenic by at least one study. In order to identify variants with high likelihood of being benign, we chose 4 conservative amino acid variants at positions that are not conserved among human paralogs, which are solvent exposed in our model, and with their side-chain extending into solvent.

For genomic variants, the protein coding effect was annotated by SnpEff [71]. Protein variants are often reported in the literature, but without mention of the exact DNA change that produced them. In order to be comprehensive, when beginning from an amino acid change, we identified all DNA changes that could have generated it. Each was annotated by SIFT [16], PolyPhen2 [17], and MetaLR [72] predictions, CADD [73] scores, and allele frequencies from ExAC [70] and 1000Genomes. When differences in annotations were present for a given amino acid change, the DNA change with the most damaging predicted effect was utilized.

### Molecular dynamics simulations

Our model was energy minimized for 5000 steps of steepest descent followed by 5000 steps of adaptive conjugate gradient, enforcing a maximum root-mean-square derivative convergence criteria of 1.0 and 0.2 kcal mol^-1^ Å,^-1^ respectively. The minimized TGFBR2 kinase model was refined by a 2ns molecular dynamics (MD) simulation using the CHARMm c36b2 all-atom force-field at a temperature of 300 K [74] and a 2fs time step. The molecule was first energy minimized using steepest descent followed by conjugant gradient and the SHAKE [75] procedure. A distance-dependent implicit solvent model was used with a dielectric constant of 80 and a pH of 7.4. Conformations from each simulation were saved every 20ps for further analyses. RMSD values were reported for each after aligning to the initial conformation. RMSF values were calculated at the residue level across trajectories aligned to the initial WT conformation. Alpha-carbon coordinates from all simulations are available as a supplemental data file.

### Monitoring structural features

Docking of the adenine molecule was equivalent in both potential template structures; ACVR2B and ACVR2A. Thus, adenine was docked into the TGFBR2 model in a similar manner to what is found in both template structures by superimposing the template proteins with our model and comparing the position of the bound ligands. Intermolecular interactions of the TGFBR2 Kinase-Adenine complex including salt bridge interactions, hydrogen bonds, electrostatic interactions, and hydrophobic interactions were calculated in the Receptor-Ligand Function of Discovery Studio version 4.5 [55]. Folding stability changes upon mutation, measured by ΔΔG_fold_, were computed using FoldX [76, 77] version 4.

We monitored the dynamics of the ATP binding site using three vectors within the protein. These consisted of the instantaneous distance between C^α^ atoms of residues around the ligand-binding site: F327 “above” the ligand, L386 below, and F255 “across from” the ligand in the p-loop. We also measured the distance from each of these three points to the C^5^ atom of the adenine molecule. Together, these six distances were used to define the shape of the ligand binding site and the position of the adenine within. Sequence comparison with other kinases helped us to define the boundaries of the TGFBR2 activation loop with its characteristic N-terminal DFG and C-terminal MAP motifs. Recent studies [15] have shown that the separation between the center of mass (COM) of residues nearby the αC-helix and within the activation loop can distinguish between activated and inactivated conformations. We have also monitored these distances across our simulations.

## Supporting information

S1 Data Data file containing alpha-carbon coordinates from the MD simulations used in this analysis. This data file contains structured coordinates for each alpha carbon from each simulation. As our analyses primarily utilized alpha-carbon positions for calculation, this file contains the minimal data required to reproduce our analysis.
(GZ) Click here for additional data file.

S1 Fig Annotated MSA of TGFβR2 paralogs. Secondary structure elements from our model are shown above the MSA and color coded ConSurf levels below. Sequences within the MSA are colored by physicochemical properties using JalView, and scaled in their intensity such that residues within columns that are not conserved (< 20% identity) are not colored. Residue numbering is according to TGFβR2. Regions where paralogs have insertions relative to TGFβR2 are indicated by a blue line and blue wedge above the MSA.
(PNG)

S2 Fig Heatmap of hydrogen bond occupancies. **A)** For each variant, we calculate the occupancy of each hydrogen bond pair at the residue level and display pairs that have at least 50% occupancy in at least 1 simulation. A residue pair is considered to be interacting if any atoms within them are involved in hydrogen bonding defined geometrically by a maximum distance of 3.2 Å and a 30° D-H-A angle using the HBonds VMD plugin. **B)** The subset of residue pairs where ≤ 5 simulations exhibit occupancy of ≤ 0.25 is shown. The checkered pattern of which cells correspond to hydrogen bonded pairs that are typically present with moderate to high occupancy, but which are lost for specific variants.
(PNG)

S3 Fig Upon mutation to A, D, or E, residue 277 completely loses hydrogen bond contacts with D397 and E290. The protein is colored as in Fig 1, with carbon atoms colored the same as the associated cartoon representation. Side chain nitrogen atoms are colored dark blue and oxygen red. Hydrogen atoms are omitted for simplicity.
(PNG)

S4 Fig Relationship between active site markers and the bound ligand. **A)** WT and the C394W pathogenic variant are shown as examples; similar to Fig 3. **B)** Benign variants are added and superimpose on the WT values. **C)** All 30 pathogenic variants studied here are included. They demonstrate a considerable spread, indicating that some have a substantial effect on ligand orientation, while others exhibit WT-like binding. Additionally, there are two patters to ligand-escape: one that is C394W-like and a second in the opposite direction.
(PNG)

S5 Fig Comparison between our homology-based model (blue) and the recently published crystal structure (orange). Regions not resolved in the crystal structure are colored white. Regions exhibiting relatively large deviation are colored in lighter tones. Ignoring these regions, the structures are extremely similar; 1.295 Å C^α^ RMSD. Adenine’s general positioning is identical, but the orientation is rotated ~60° to position the 5-member ring facing towards the activation loop. In the crystal structure, five charged amino acids were mutated to alanine and are marked by spheres at their C^α^ atoms.
(PNG)
