# Supplementary figures and images for: Molecular modeling and molecular dynamic simulation of the effects of variants in the TGFBR2 kinase domain as a paradigm for interpretation of variants obtained by next generation sequencing
Source: PLoS One. 2017 Feb 9;12(2):e0170822. doi: 10.1371/journal.pone.0170822 (PMC5300139; doi:10.1371/journal.pone.0170822)

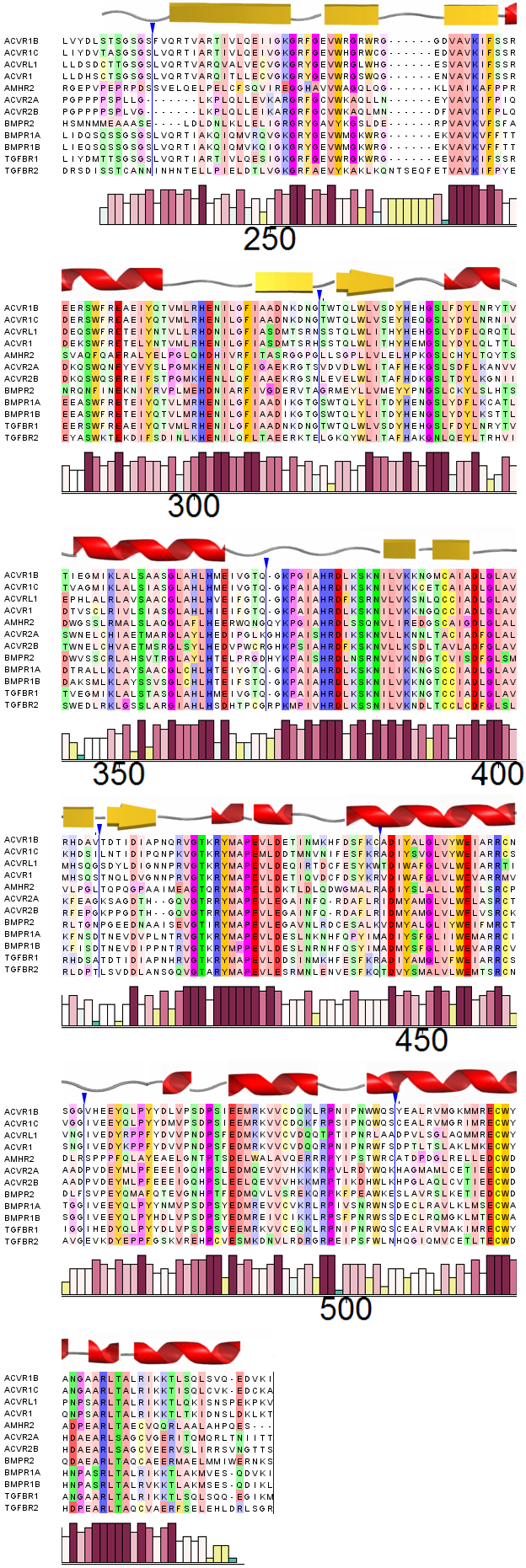

Supplement: S1 Fig — Secondary structure elements from our model are shown above the MSA and color coded ConSurf levels below. Sequences within the MSA are colored by physicochemical properties using JalView, and scaled in their intensity such that residues within columns that are not conserved (< 20% identity) are not colored. Residue numbering is according to TGFβR2. Regions where paralogs have insertions relative to TGFβR2 are indicated by a blue line and blue wedge above the MSA. (PNG) [file pone.0170822.s002.png]

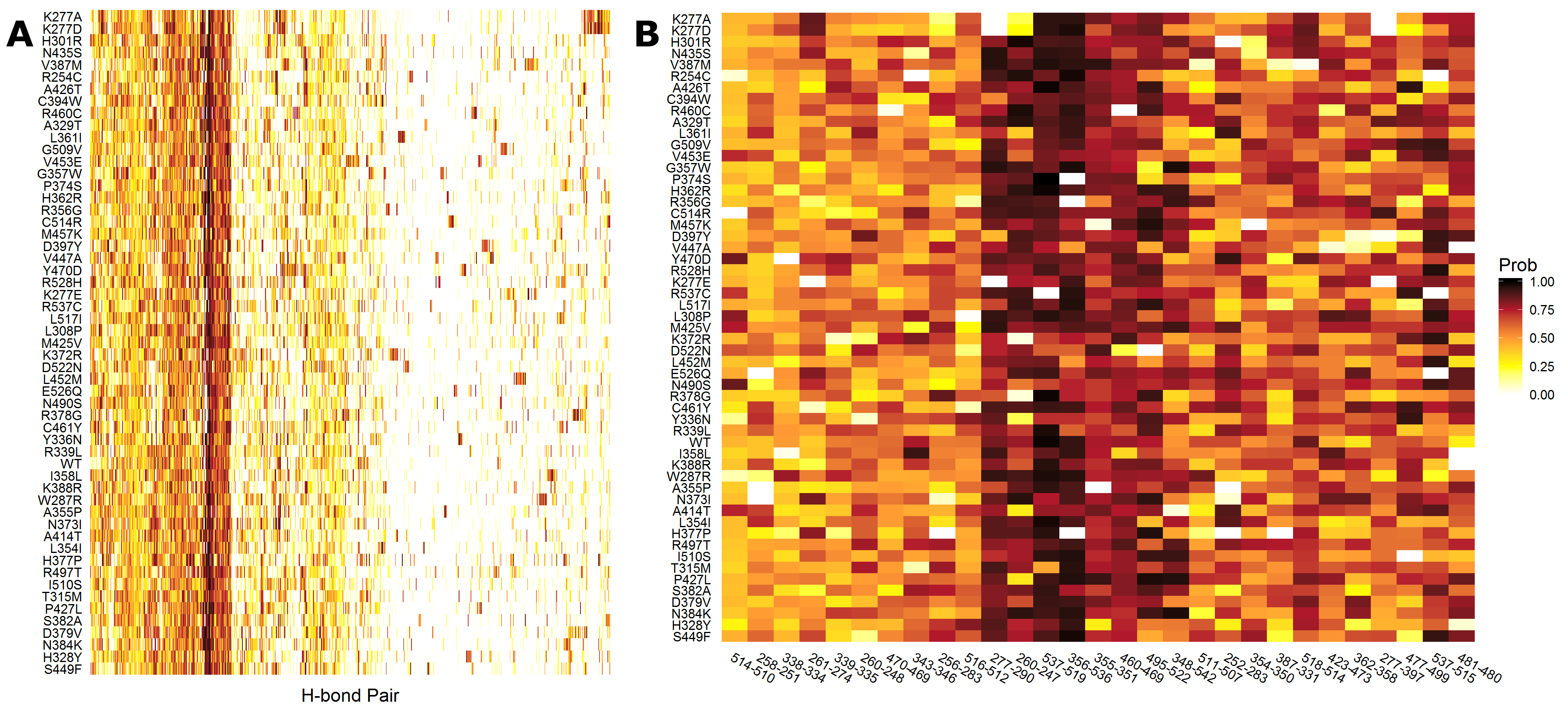

Supplement: S2 Fig — A) For each variant, we calculate the occupancy of each hydrogen bond pair at the residue level and display pairs that have at least 50% occupancy in at least 1 simulation. A residue pair is considered to be interacting if any atoms within them are involved in hydrogen bonding defined geometrically by a maximum distance of 3.2 Å and a 30° D-H-A angle using the HBonds VMD plugin. B) The subset of residue pairs where ≤ 5 simulations exhibit occupancy of ≤ 0.25 is shown. The checkered pattern of which cells correspond to hydrogen bonded pairs that are typically present with moderate to high occupancy, but which are lost for specific variants. (PNG) [file pone.0170822.s003.png]

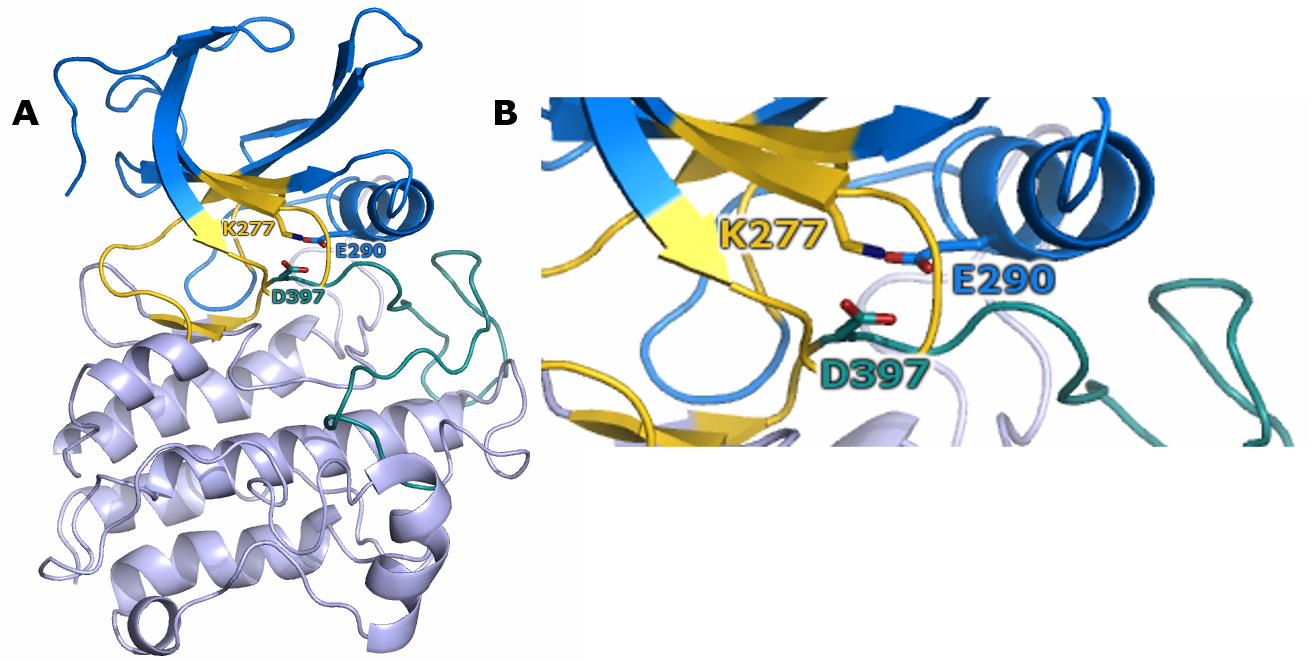

Supplement: S3 Fig — The protein is colored as in Fig 1, with carbon atoms colored the same as the associated cartoon representation. Side chain nitrogen atoms are colored dark blue and oxygen red. Hydrogen atoms are omitted for simplicity. (PNG) [file pone.0170822.s004.png]

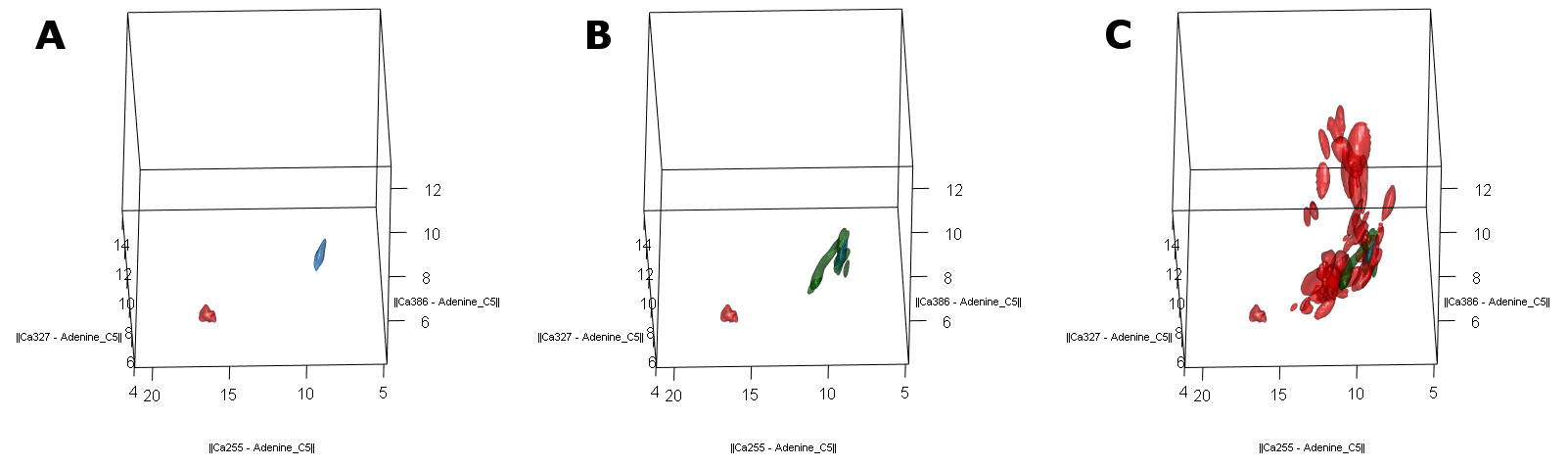

Supplement: S4 Fig — A) WT and the C394W pathogenic variant are shown as examples; similar to Fig 3. B) Benign variants are added and superimpose on the WT values. C) All 30 pathogenic variants studied here are included. They demonstrate a considerable spread, indicating that some have a substantial effect on ligand orientation, while others exhibit WT-like binding. Additionally, there are two patters to ligand-escape: one that is C394W-like and a second in the opposite direction. (PNG) [file pone.0170822.s005.png]

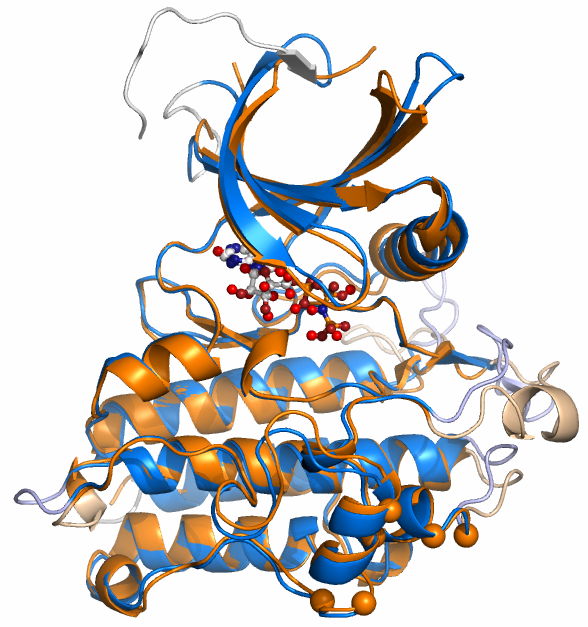

Supplement: S5 Fig — Regions not resolved in the crystal structure are colored white. Regions exhibiting relatively large deviation are colored in lighter tones. Ignoring these regions, the structures are extremely similar; 1.295 Å Cα RMSD. Adenine’s general positioning is identical, but the orientation is rotated ~60° to position the 5-member ring facing towards the activation loop. In the crystal structure, five charged amino acids were mutated to alanine and are marked by spheres at their Cα atoms. (PNG) [file pone.0170822.s006.png]
